# Testing Multi-Frequency Low-Cost GNSS Receivers for Geodetic Monitoring Purposes

**DOI:** 10.3390/s20164375

**Published:** 2020-08-05

**Authors:** Veton Hamza, Bojan Stopar, Tomaž Ambrožič, Goran Turk, Oskar Sterle

**Affiliations:** Faculty of Civil and Geodetic Engineering, University of Ljubljana, Jamova Cesta 2, 1000 Ljubljana, Slovenia; bojan.stopar@fgg.uni-lj.si (B.S.); tomaz.ambrozic@fgg.uni-lj.si (T.A.); goran.turk@fgg.uni-lj.si (G.T.); oskar.sterle@fgg.uni-lj.si (O.S.)

**Keywords:** monitoring, low-cost instrumentation, displacements, GNSS

## Abstract

Global Navigation Satellite System (GNSS) technology is widely used for geodetic monitoring purposes. However, in cases where a higher risk of receiver damage is expected, geodetic GNSS receivers may be considered too expensive to be used. As an alternative, low-cost GNSS receivers that are cheap, light, and prove to be of adequate quality over short baselines, are considered. The main goal of this research is to evaluate the positional precision of a multi-frequency low-cost instrument, namely, ZED-F9P with u-blox ANN-MB-00 antenna, and to investigate its potential for displacement detection. We determined the positional precision within static survey, and the displacement detection within dynamic survey. In both cases, two baselines were set, with the same rover point equipped with a low-cost GNSS instrument. The base point of the first baseline was observed with a geodetic GNSS instrument, whereas the second baseline was observed with a low-cost GNSS instrument. The results from static survey for both baselines showed comparable results for horizontal components; the precision was on a level of 2 mm or better. For the height component, the results show a better performance of low-cost instruments. This may be a consequence of unknown antenna calibration parameters for low-cost GNSS antenna, while statistically significant coordinates of rover points were obtained from both baselines. The difference was again more significant in the height component. For the displacement detection, a device was used that imposes controlled movements with sub-millimeter accuracy. Results, obtained on a basis of 30-min sessions, show that low-cost GNSS instruments can detect displacements from 10 mm upwards with a high level of reliability. On the other hand, low-cost instruments performed slightly worse as far as accuracy is concerned.

## 1. Introduction

The natural as well as built environment is continuously subjected to various forces in nature that may cause alterations in the shape and size of the environment. In some cases, those deformations may lead to damage and safety-of-life hazards. In order to prevent the worst-case scenario, manmade structures and natural hazards are monitored with geodetic sensors and methods on a basis of repeated geodetic surveys [1,2]. Traditionally, sensors and methods of terrestrial geodesy have usually been applied, however, with the availability and development of satellite geodesy, especially Global Navigation Satellite Systems (GNSS); the latter has also been recognized as a methodology for deformation monitoring [3,4,5].

The possibility to work continuously, remotely, in real-time, or with post-processing has resulted in a wide use of GNSS technology for geodetic monitoring [3,4]. In general, high-precision, but expensive GNSS receivers are used to determine 3D displacements. In cases of different construction objects, such as bridges, dams, and viaducts, the geodetic GNSS receivers have been shown to be suitable in terms of accuracy and have been classified as proper sensors for geodetic monitoring [3,4,5,6,7,8,9]. A study in [5] emphasized that geodetic receivers were accurate enough for estimating displacement in case of dam monitoring, however, compared to the pendulum, where a long observation period is considered, geodetic GNSS receivers were inferior.

Although geodetic receivers meet the quality criteria for monitoring tasks, their high price calls their use into question where there is a higher risk of instrument damage. An alternative solution may be seen in using low-cost GNSS receivers that have been shown to achieve high accuracy over short baselines [10,11,12,13,14].

### 1.1. Overview

In monitoring natural hazards and manmade structures, various geodetic methods may be used. Low-cost GNSS technology provides an inexpensive and flexible solution for geodetic monitoring purposes, especially when open-source software is considered [13,15]. Moreover, the GNSS data are reliable enough to be combined with other sensors to build up an early warning system for monitoring purposes [16].

The current number of scientific research regarding low-cost GNSS receivers is very limited. Only a few papers have been published that have investigated the potential and applicability of the low-cost receivers for geodetic monitoring purposes. In general, the performance of single-frequency, low-cost GNSS receivers was tested and analyzed in open-sky environments with reduced multipath impact. Low-cost GNSS receivers were usually set up as rover stations, whereas the geodetic, low-cost receiver, or virtual reference station (VRS) were used as base stations [11,12,14,17]. Studies showed that results based on low-cost GNSS receivers were promising for both static and kinematic mode [12,17]. Biagi et al. [12] tested the accuracy of the single-frequency u-blox NEO-7P receivers and the results indicated that horizontal displacements of 15 mm can be detected when a geodetic receiver is used as a base station. On the other hand, the results on a basis of u-blox NEO-7P receivers only (both base station and rover) were less accurate, but still satisfactory considering that few centimeter movements are expected in monitoring natural hazards. Two-dimensional displacements in range of 25 mm and 3D displacements in range of 35 mm were detected by low-cost receivers only. Caldera et al. [11] analyzed the performance of u-blox EVK-6T receivers in open-sky, for short baselines, using only GPS data. The results indicated that 3D displacements on a level of few millimeters can be detected in daily sessions. Using VRS as a base station reduces baseline length and, consequently, the positional accuracy is improved. Cina and Piras [14] analyzed baseline with VRS as base station and low-cost u-blox EVK-5T receiver in combination with a geodetic external antenna as a rover and obtained positional accuracy on a sub-cm level.

NEO-M8P, a single-frequency receiver, was used with Trimble Bullet 360° antenna for monitoring real-time kinematic (RTK) and post-processing mode, where GNSS data were processed with open-source software and the entire monitoring station was built with low costs [13]. RTK positional accuracy was on a level of 4 and 8 mm for the horizontal and vertical component, respectively. The results were further improved with post-processing and the accuracy for hourly sessions was estimated to be 2 and 5 mm for horizontal and vertical component, respectively [13]. The same single-frequency receiver was also tested by Garrido-Carretro et al. [18] in RTK mode with low-cost antennae over short baseline (350 m). The results using single-base RTK solution indicate accuracy of 5.5 mm for the horizontal component and 11 mm for the vertical component.

Nowadays, multi-frequency, low-cost GNSS receivers are also available at the market. This type of GNSS receiver can receive satellite signals in both L1 and L2 frequencies from all available satellite constellations. Gebre-Egziaber [10] tested multi-frequency receiver Piksi Multi and the mid-range receiver Eclipse P307 in static and dynamic applications. Both receivers performed well compared with the results obtained from the geodetic receiver, used as a reference. ZED-F9P GNSS module in combination with a patch antenna reached centimeter accuracy in 3D positioning. Similar results were obtained when an external Trimble R10 antenna was applied [19]. Lastly, low-cost dual-frequency receivers in combination with different antennas were used by Krietemeyer et al. [20] for the estimation of Zenith Tropospheric Delay (ZTD). When relative calibration parameters for antennae were applied, the results indicated that the ZTD was estimated with a comparable accuracy with results obtained from the geodetic antennas. To date, mostly single-frequency low-cost receivers were used for displacement detection or geodetic monitoring, while in this study we tested the potential of multi-frequency low-cost receivers for displacements detection which proved satisfactory performance in open sky.

### 1.2. Objective and Organization of the Work

In this study, a multi-frequency low-cost receiver ZED-F9P and u-blox GNSS antenna ANN-MB-00 were analyzed. GNSS data were obtained in favorable conditions in open-sky environment where reduced impact of multipath is expected. The main objective is to evaluate the positional quality of the low-cost receivers and to identify the minimum level of displacements that can be detected with such inexpensive instruments.

The paper is structured as follows: firstly, an overview of the usage of single- and double-frequency, low-cost receivers for geodetic monitoring is described (Section 1). Then, the configuration and connection of the simpleRTK2B board with OpenLog are shown. Basic statistics are adopted to evaluate the positional quality of ZED-F9P, hypotheses are tested to define the range of displacements that can be detected (Section 2). Obtained results are presented and discussed (Section 3). Lastly, conclusions and remarks are presented (Section 4).

## 2. Material and Methods

### 2.1. Low-Cost GNSS Receiver ZED-F9P

Nowadays, different types of multi-frequency GNSS receivers that are cheap, light, and able to work in static and dynamic applications are available. At the beginning of 2019, the u-blox company launched the ZED-F9P chip that can receive satellite signals in the lower and upper-L band (L1C/A, L1OF, E1, B1l, L2C, L2OF, E5b, B2l) from all available satellite constellations (GPS, GLONASS, Galileo, BeiDou) [21]. The u-blox integrated the ZED-F9P module in C099-F9P application board that can work in RTK mode and transmit RTCM corrections via cable, Wi-Fi, or Bluetooth and is easily configurable through the open-source evaluation software u-center [21,22]. Another board that houses the ZED-F9P chip is simpleRTK2B offered by Ardusimple. The board and u-blox multiband antenna ANN-MB-00 can be purchased with low cost (270 EUR). SimpleRTK2B can be also configured by u-center and has the same GNSS chip with an integrated RTK engine, which enables coordinate estimation in real-time. Both boards that own a ZED-F9P chip may be a proper solution for geodetic monitoring of natural hazards, however, they should first be tested in an optimal environment to analyze their performance.

### 2.2. SimpleRTK2B Board and OpenLog Connection

**Figure 1:**
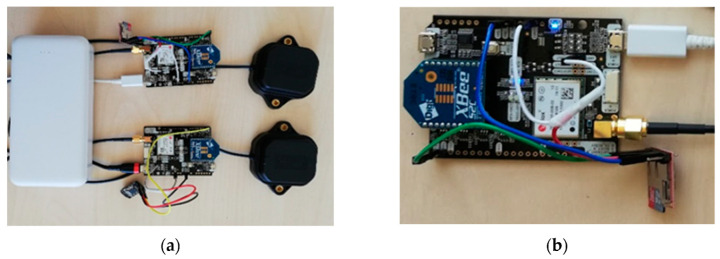
SimpleRTK2B boards and other used equipment: (**a**) SimpleRTK2B boards connected to ANN-MB-00 antennae and power bank for power supply; (**b**) Connection of SimpleRTK2B board with OpenLog DEV-13955.

Two SimpleRTK2B boards together with u-blox GNSS antennas ANN-MB-00 were used in our case study (Figure 1a). In the initial phase of the experiment, simpleRTK2B boards were connected with the laptop, where the USB port was used for power supply as well as for data storage. However, it turned out that the laptop was not an adequate solution, since its battery ran out after three hours. The bad weather conditions were also not a positive factor for using laptop. Instead, we used Sparkfun’s OpenLog DEV-13955 for data storage. It was connected to the simpleRTK2B with jumper wires (Figure 1b). The OpenLog can send all messages that come from RX1/TX1 pin to the mounted MicroSD. The TX1 pin of the board needs to be connected with the RX1 pin of the OpenLog to transmit messages, 5V_OUT pin, and the IOREF pin was connected with the VCC pin to define the voltage and the GND pin to GND pin [23,24]. OpenLog can work with MicroSD that has a memory capacity from 512 MB up to 32 GB. Two lights are flashing during operation, one continuously for communication, and the other one when the OpenLog records data in MicroSD [24].

The boards were configured with the u-center evaluation software to output messages through the UART1 port. The baud rate was set to 115,200—the recommended value for the 9th generation of u-blox GNSS receivers. To get observations in RINEX format, RXM-RAWX and RXM-SFRBX messages were enabled in UART1. The data were stored in the UBX file that was converted to RINEX by using the modified version (demo5_b33b) of the RTKLIB. Since the version of RTKLIB developed by Tomoji Takasu had difficulties converting the UBX file to the RINEX file, we decided to use the modified version developed by Tim Everett that is used for converting UBX files and data processing [25,26]. To ensure power supply, two power banks Platinet PMPB20TW with 20,000 mAh^5V^ capacity were used. The energy consumption of the SimpleRTK2B, antenna, and OpenLog is pretty low (215 mAh in total); the survey can therefore last for approximately 90 h.

### 2.3. Study Area

**Figure 2:**
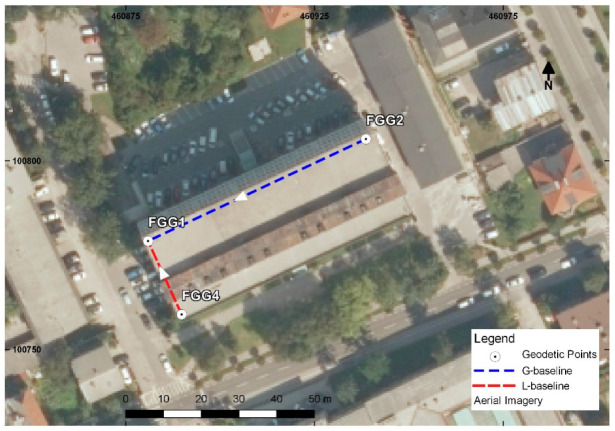
Faculty of Civil and Geodetic Engineering building.

The roof of the Faculty of Civil and Geodetic Engineering—University of Ljubljana (UL FGG) building was selected as the location for the experiment (Figure 2). The roof represents generic conditions and is equipped with concrete pillars that enable force centering of GNSS instruments. The iron plates on the top of the pillars may be seen as a ground plane during the observations, which is recommended in the case of patch antennas [27]. On the other hand, the coordinates of the pillars are known, as they were determined from several longer static GNSS surveys in the past. Low-cost antennas are very sensitive to multipath error and additionally, phase center offset and variations are unknown. The latter has an impact on the results, especially in the height component [12]. To mitigate the multipath error, an open-sky environment was selected (roof of the faculty), while the missing antenna calibration issue will be addressed in the future.

**Figure 3:**
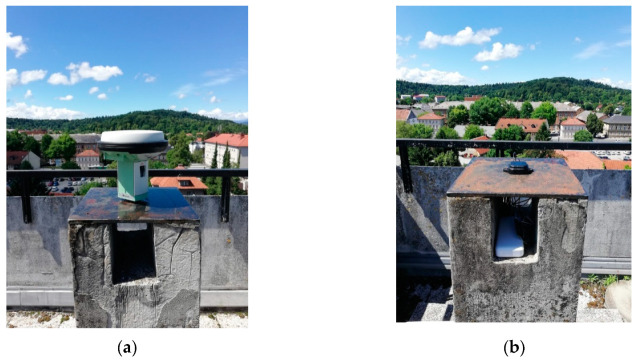
GNSS receivers: (**a**) Reference station Leica GS 15 in pillar FGG2; (**b**) Rover ZED-F9P in pillar FGG1.

Geodetic receiver Leica GS 15 with integrated antenna LEIGS15 NONE was put on the pillar FGG2 (Figure 3a), while two low-cost receivers were placed on pillars FGG1 and FGG4 (Figure 2). GNSS receiver in point FGG1 (Figure 3b) is used as a rover, where the other two are used as reference stations. Spatial distance defined between FGG2 and FGG1 is 65 m, and in the upcoming tests, we refer to this baseline as the G-baseline. The abbreviation G represents geodetic GNSS receiver Leica GS 15 (base). The second baseline, denoted as L-baseline, is 18 m long and was observed with two low-cost receivers, i.e., the base is also a low-cost receiver.

### 2.4. Evaluation Approach of ZED-F9P

The evaluation approach of the GNSS module ZED-F9P includes two aspects, namely, the positional quality and investigation of its potential for displacement detection. Firstly, static observations are carried out to analyze the reliability of GNSS data and to assess the quality of multi-frequency, low-cost instrument positioning. Secondly, controlled movements of the low-cost antenna are imposed to analyze the minimum level of displacement that may be detected with low-cost GNSS instruments.

#### 2.4.1. Positional Quality

To evaluate the positional quality of ZED-F9P, static observations were carried out at 1 Hz for 28 h from the 31st to 34th day of the year 2020 (from 31st January until 3rd February), where data from three different navigation systems, namely, GPS, GLONASS, and Galileo, were obtained. GNSS data were processed with open-source software RTKLIB (demo5_b33b) in hourly sessions. The processing parameters used in RTKLIB are shown in Table 1. The number of satellites was at least 15 in every session for both baselines. We initially used also Leica Infinity for processing of the GNSS data, but since it processed data from L1 frequency only and somehow ignored all L2 observations, we did not use it in further analysis.

The results of GNSS data processing were estimated coordinates of the FGG1 point; for each hourly session the mean value of obtained coordinates was calculated, for both baselines, the G-baseline and the L-baseline. Baseline residuals were used to obtain some elementary statistics, such as minimum and maximum value, and Root–Mean–Square–Error (RMSE). No outliers were detected in GNSS data; all residuals were in the interval less than 3σ, where the verification was done by τ-test and Tukey’s Method [28,29]. Residuals were, in most cases, less than 4 mm for all coordinate components (Y—east, X—north, and h—ellipsoidal height). To better express their differences, we followed a classification adopted from Biagi et al. [12], where five groups were defined. The first group represents all residuals in a range from 0 to 2 mm, the second from 2 to 4 mm, the third from 4 to 6 mm, the fourth from 6 to 8 mm, and the last one from 8 to 10 mm.

Because GNSS data from two baselines were processed, two triplets of FGG1 coordinates were obtained for each session. One set of coordinates belongs to the G-baseline, denoted as *Y*_G_, *X*_G_, and *h*_G_, and the other one to the L-baseline, denoted as *Y*_L_, *X*_L_, and *h*_L_. The differences of estimated 1D, 2D and, 3D positions from both (L- and G-) baselines are determined as

(1) $$b_{1}=h_{L}-h_{G}$$

(2) $$b_{2}=\sqrt{{\left(Y_{L}-Y_{G}\right)}^{2}+{\left(X_{L}-X_{G}\right)}^{2}}$$

(3) $$b_{3}=\sqrt{{\left(Y_{L}-Y_{G}\right)}^{2}+{\left(X_{L}-X_{G}\right)}^{2}+{\left(h_{L}-h_{G}\right)}^{2}}$$

The differences in estimated coordinates from Equations (1)–(3) are mainly due to different GNSS instruments at both base stations, the geodetic GNSS receiver/antenna at FGG2 and low-cost GNSS receiver/antenna at FGG4 [12]. To compare the performance of different GNSS instruments at both base stations, the analysis of variance (ANOVA) is applied separately for each coordinate component (*Y*, *X*, and *h*). ANOVA is used to identify if there is a significant difference between the means of two groups, i.e., means of FGG1 coordinates obtained from both baselines [30]. The comparison of coordinate means is done by defining the following hypotheses:

H_0_: The use of different types of GNSS instruments doesn’t affect coordinates;

H_a_: The use of different types of GNSS instruments affects coordinates.

Results of the positional analysis are presented in the first part of Section 3, namely, in Section 3.1.

#### 2.4.2. Displacement Detection

**Figure 4:**
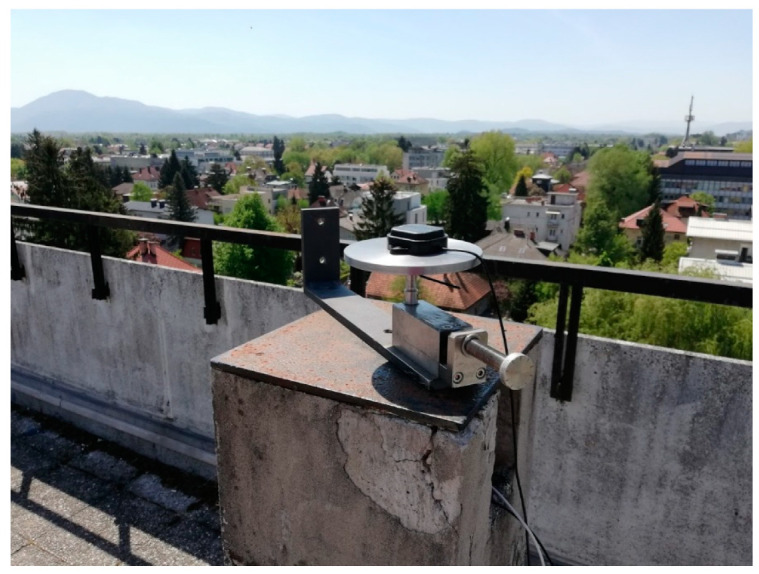
Specially constructed device for control movements and ground plane.

To analyze the possibility of displacement detection, a mechanical device (Figure 4) that can impose controlled movements with sub-millimeter accuracy in the horizontal plane was used. The rover antenna was placed on a specially designed iron ground plane with a 15-cm diameter mounted on the device (Figure 4), which has already been proved to improve the results of the survey with low-cost antennae [20].

Measurements were performed from 52nd until 55th day of the year 2020 (from 21st February until 24th February), with sampling rate set to 1 Hz. The survey consisted of a series of 30-min sessions, where the rover was moved by 5 mm in-between two consecutive sessions. All movements were imposed in horizontal plane only. According to Günter et al. [31], 30-min sessions can be considered as suitable for detecting displacements. The same survey was then repeated for all the following days and, as a result, 270 displacements, classified into 10 groups, were obtained. The first group represents a displacement of 5 mm, the second a displacement of 10 mm, the third of 15 mm, and so on. The last, 10th, group is represented with a displacement of 50 mm. Consecutive groups are therefore mutually different for 5 mm, from 5 up to 50 mm. This classification is done to analyze the minimum value of displacements which can be detected by using only low-cost instruments or low-cost instruments in combination with geodetic receivers on base station.

In order to determine if the displacements of the rover are significant, statistical tests are applied [28,32,33,34]. To test point stability between two different sessions, the following hypotheses are used:

H_0_: d=0; Point remained stable between two sessions;

H_a_: d≠0; Point did not remain stable between two sessions—the displacement is detected.

The test statistic that is used to verify the rejection of the null hypothesis is defined as follows [34]

(4) $$T=\frac{d}{\sigma_{d}}\;$$

The verification of the null hypothesis is done by comparing calculated test values with the critical values. The test is dependent on the displacement dimension, i.e., for 1D, 2D, or 3D (see Equations (1)–(3)), however, the significance level of 5% (α = 0.05) was set for all cases [34]. The displacement between two sessions, session i and session j (j≠i), for all three dimensions (1D, 2D, and 3D) is as follows

(5) $$d_{1}=\Delta h=h_{j}-h_{i}$$

(6) $$d_{2}=\sqrt{\Delta Y^{2}+\Delta X^{2}}=\sqrt{{\left(Y_{j}-Y_{i}\right)}^{2}+{\left(X_{j}-X_{i}\right)}^{2}}$$

(7) $$d_{3}=\sqrt{\Delta Y^{2}+\Delta X^{2}+\Delta h^{2}}=\sqrt{{\left(Y_{j}-Y_{i}\right)}^{2}+{\left(X_{j}-X_{i}\right)}^{2}+{\left(h_{j}-h_{i}\right)}^{2}}$$

where:

$Y_{i},X_{i},h_{i}$—FGG1 coordinates from the ith session;

$Y_{j},X_{j},h_{j}$—FGG1 coordinates from the jth session.

Based on the error propagation law, the variance of the considered displacements was estimated as follows [35]

(8) $$\sigma_{d}^{2}=J_{d}\Sigma_{ij}J_{d}^{T}\;$$

Jacobi matrices $\left(J_{d}\right)$, design matrices in case of error propagation law, are defined for 1D, 2D, and 3D displacements, respectively [33]

(9) $$J_{d_{1}}=\left[\begin{array}{cc} \frac{\partial d_{1}}{\partial h_{i}} & \frac{\partial d_{1}}{\partial h_{j}} \end{array}\right]$$

(10) $$J_{d_{2}}=\left[\frac{\partial d_{2}}{\partial Y_{i}}\;\frac{\partial d_{2}}{\partial X_{i}}\;\frac{\partial d_{2}}{\partial Y_{j}}\;\frac{\partial d_{2}}{\partial X_{j}}\right]$$

(11) $$J_{d_{3}}=\left[\frac{\partial d_{3}}{\partial Y_{i}}\;\frac{\partial d_{3}}{\partial X_{i}}\;\frac{\partial d_{3}}{\partial h_{i}}\;\frac{\partial d_{3}}{\partial Y_{j}}\;\frac{\partial d_{3}}{\partial X_{j}}\;\frac{\partial d_{3}}{\partial h_{j}}\right]$$

Variance–covariance matrices $\left(\Sigma_{ij}\right)$ for all three dimensions have the following form [33]

(12) $$1D:\;\Sigma_{ij}=\left[\begin{array}{cc} \sigma_{h_{i}}^{2} & 0\\ 0 & \sigma_{h_{j}}^{2} \end{array}\right]$$

(13) $$2D:\;\Sigma_{ij}=\left[\begin{array}{cccc} \sigma_{Y_{i}}^{2} & \sigma_{Y_{i}X_{i}} & 0 & 0\\ \sigma_{Y_{i}X_{i}} & \sigma_{X_{i}}^{2} & 0 & 0\\ 0 & 0 & \sigma_{Y_{j}}^{2} & \sigma_{Y_{j}X_{j}}\\ 0 & 0 & \sigma_{Y_{j}X_{j}} & \sigma_{X_{j}}^{2} \end{array}\right]$$

(14) $$3D:\;\Sigma_{ij}=\left[\begin{array}{cccccc} \sigma_{Y_{i}}^{2} & \sigma_{Y_{i}X_{i}} & \sigma_{Y_{i}h_{i}} & 0 & 0 & 0\\ \sigma_{Y_{i}X_{i}} & \sigma_{X_{i}}^{2} & \sigma_{X_{i}h_{i}} & 0 & 0 & 0\\ \sigma_{Y_{i}h_{i}} & \sigma_{X_{i}h_{i}} & \sigma_{h_{i}}^{2} & 0 & 0 & 0\\ 0 & 0 & 0 & \sigma_{Y_{j}}^{2} & \sigma_{Y_{j}X_{j}} & \sigma_{Y_{j}h_{j}}\\ 0 & 0 & 0 & \sigma_{Y_{j}X_{j}} & \sigma_{X_{j}}^{2} & \sigma_{X_{j}h_{j}}\\ 0 & 0 & 0 & \sigma_{Y_{j}h_{j}} & \sigma_{X_{j}h_{j}} & \sigma_{h_{j}}^{2} \end{array}\right]$$

According to 1D test statistic from Equations (1), (9) and (12), the statistic is distributed with the standardized normal distribution. On the other hand, the 2D test statistic (Equations (2), (10) and (13)) and 3D test statistic (Equations (3), (11) and (14)) are distributed with χ^2^ distribution with 2 and 3 degrees of freedom [34].

Since the imposed displacements of the rover antenna were in the horizontal plane only, we expect the test statistic for all 1D displacement to be smaller than its critical value. To test if the ellipsoidal height has changed between two sessions, the following hypotheses are set:

H_0_: $d_{1}=0$; Point didn’t move vertically between two sessions;

H_a_: $d_{1}\neq 0$; Point moved vertically between two sessions—the vertical displacement is detected.

Rejection of the null hypothesis is verified based on the test statistic and critical value (*t*_critical_ = 1.96) determined from standardized normal distribution [34]. The null hypothesis was tested 720 times in total, for all possible pairs of sessions. The number of tests is much higher for 1D compared to both of the following cases (2D and 3D tests) because observations from positional quality assessment surveys (data from Section 2.4.1) were also used.

The horizontal coordinates of point FGG1 were also tested in order to detect horizontal displacement. During the survey, 270 possible displacements were imposed, which ranged from 5 to 50 mm. Differences between sessions of two identical positions were not tested. In this case, we are testing only differences that correspond to truly imposed displacements and may, therefore, determine the minimum level of the displacement for low-cost GNSS instruments. The following hypothesis are set:

H_0_: $d_{2}=0$; Point didn’t move horizontally between two sessions;

H_a_: $d_{2}\neq 0$; Point moved horizontally between session—the horizontal displacement is detected.

Verification of rejection of the null hypothesis is done by comparing test statistics with the critical value (*t*_critical_ = 2.45), determined from χ^2^ distribution with two degrees of freedom. The final critical value is determined as a square root of theoretical χ^2^ critical value.

In reality, the displacements of points are expected to be spatial (in 3D) in any direction. In this case, for 3D displacements, the following hypotheses are set:

H_0_: $d_{3}=0$; Point did not move spatially between two sessions;

H_a_: $d_{3}\neq$ 0; Point moved spatially between two sessions—the spatial displacement is detected.

**Figure 5:**
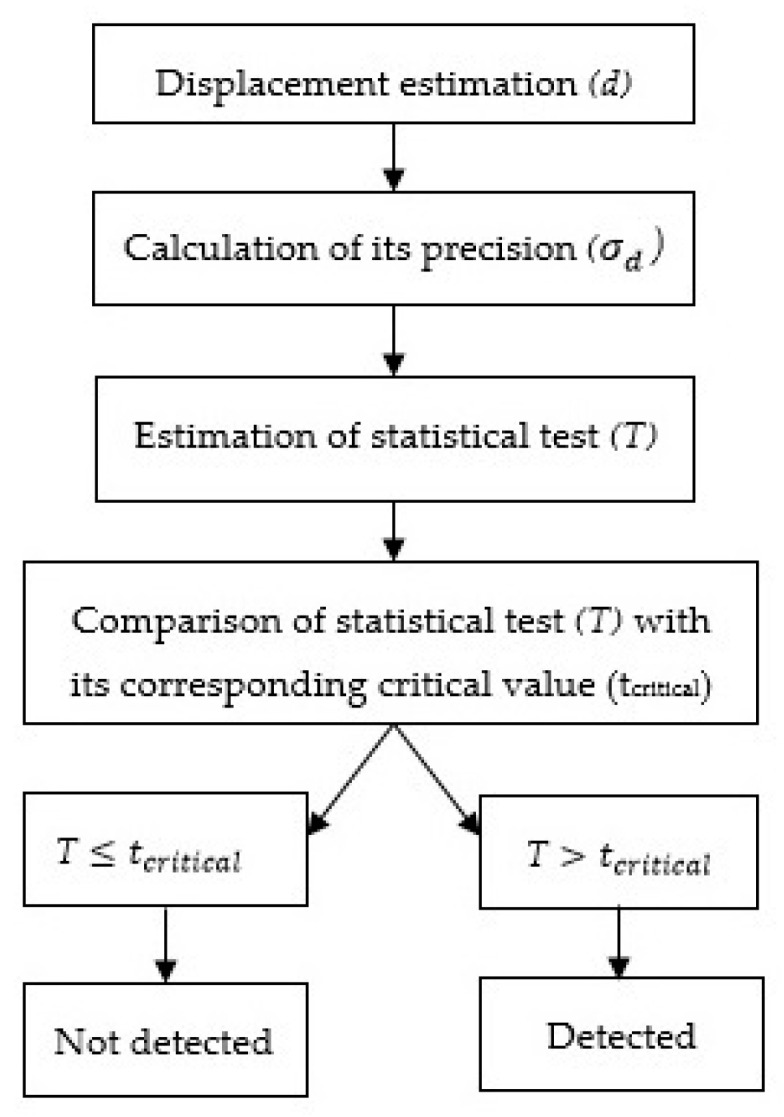
Displacement detection.

As in the case of 2D, 270 movements were imposed in total, where the defined critical value (*t*_critical_ = 2.80) is used to verify the rejection of tested hypotheses. This was again obtained from χ^2^ distribution with three degrees of freedom (and again a square root value). The procedure of displacements’ detection is shown graphically in Figure 5.

A mechanical device that imposes displacements provides them with sub-millimeter accuracy, and therefore true values of all displacements are known. In the horizontal plane, the displacements are in steps of 5 mm, from 0 to 50 mm, whereas in vertical directions there are truly no displacements, all their values are equal to 0 mm. For all estimated displacements (1D, 2D, and 3D), we also estimated Mean Absolute Error (MAE). In this way, important information will be acquired, namely, the level of accuracy of estimated displacements with respect to their true values that may be achieved with low-cost GNSS receivers. The MAE values will be determined for both baselines, for the G-baseline as well as for the L-baseline. The results are presented in the second part of the following Section 3.

## 3. Results and Discussions

This section represents the results of the methods defined in the previous section. The first part shows the assessment of the positional quality of low-cost GNSS receivers, whereas the second part represents displacement detection. In both cases, results will be presented for G-baseline as well as for L-baseline.

### 3.1. Positional Quality Assessment

Some elementary statistics for baseline residuals are presented in Table 2 (G-baseline) and Table 3 (L-baseline).

The RMSE values for both baselines, the G- and L-baseline, are comparable and are on a level of 2 mm or better for both horizontal components. However, for the vertical component, the RMSE value is slightly higher, especially for the G-baseline. Results show that in the case of L-baseline, the height component is determined with higher precision, as in the case of G-baseline. The reason may be found in unknown antenna phase center offset and variation in the low-cost GNSS antenna. Since the L-baseline has low-cost GNSS antennae for the base as well as for the rover, the bias of the missing antenna calibration is eliminated with single differences. On the other hand, for the G-baseline, which has a geodetic GNSS antenna, with known antenna calibration parameters, the missing calibration parameters affect the final results, especially the height component [12,20].

**Figure 6:**
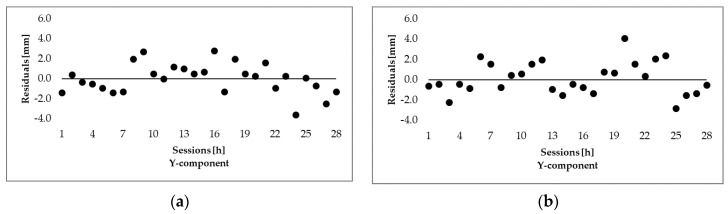

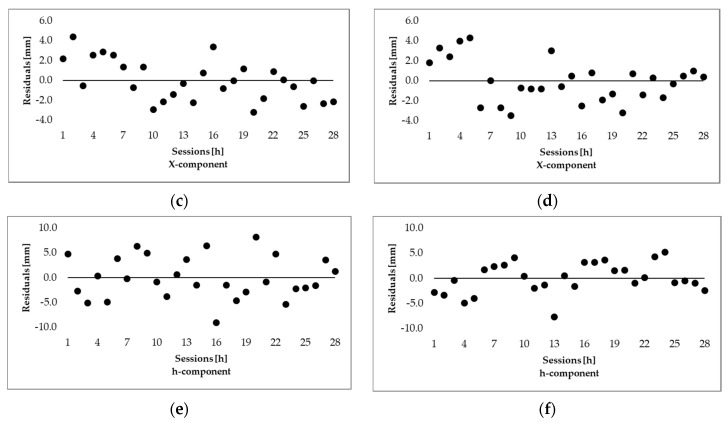
Baseline residuals for all coordinate components and both baselines: (**a**) *Y* component, G-baseline (**b**) *Y* component, L-baseline (**c**) *X* component, G-baseline (**d**) *X* component, L-baseline (**e**) height, G-baseline (**f**) height, L-baseline.

Figure 6 represents results from Table 2 and Table 3 in graphical form. Graphs in Figure 6 depicts baseline residuals for all coordinate components (Y, X, and h) and, for both baselines, the left graphs are for G-baseline, whereas the right graphs are for L-baseline.

All residuals were classified into five groups according to their absolute values, where the results are represented in Table 4 for G-baseline and in Table 5 for L-baseline. In the case of G-baseline, the residuals for both horizontal components are within 4 mm; only 3% of X residuals are in the third group. For the height component, 86% of them are within 6 mm, while the rest are equally represented in the 4th and 5th group. On the other hand, the results for the L-baseline are slightly worse, 3% of Y residuals and 6% of X residuals are in the third group. However, for the height component, the results are slightly better; all residuals are within 8 mm. This may again be due to the unknown antenna offset and phase variation bias in the case of low-cost antennae.

Differences in determined FGG1 coordinates from both tested baselines, namely, the G-baseline and L-baseline, are shown in Table 6. Differences in position arise mostly from the height component (10.4 mm), whereas the horizontal difference in position is only 2.6 mm. The difference in 3D position has been estimated with lower precision (5.2 mm), followed by 1D position (4.6 mm) while the difference in 2D position was estimated with higher precision (2.5 mm).

Table 7 represents the results of the ANOVA test, where the differences from Table 6 are analyzed. ANOVA test was performed with a significance level of 5%. The results of the test show that the null hypothesis was rejected for all three components and may confirm that using different GNSS instrument types (geodetic vs. low-cost) will significantly affect estimated baseline components [12]. The difference is again much larger for the height component. Again, the reason for this may be in uncalibrated low-cost GNSS antenna.

### 3.2. Displacement Detection Results

Within this section, the results of displacement detections are represented. Statistical tests were performed to detect displacements in height component (1D tests), where the results are shown in Table 8, in horizontal plane (2D tests), where the results are represented in Table 9 and for spatial displacements (3D tests), where the results are represented in Table 10.

Because the imposed displacements were in the horizontal plane only, we should fail to reject the null hypothesis in all cases of statistical tests for 1D displacement detection. The results from Table 8 show that this is true in 98% cases for the G-baseline but only in 77% cases for the L-baseline. This result is interesting; it shows that the height component is determined with higher accuracy in the case of the G-baseline compared to the L-baseline when the true value of zero displacement is considered. In the precision determination, the RMSE of the L-baseline was shown to be smaller than the G-baseline (see previous Section 3.1), while in detecting 1D displacements the height component obtained from G-baseline was more accurate, as shown in Table 8. The reason for these results may be found in missing antenna calibration parameters for low-cost antennae, but the performance of low-cost receivers and antennae may also account for that.

In the case of horizontal displacements, Table 9 presents the number of successfully determined displacements of size 10, 15, and 20 mm. The results for 5 mm displacements are not shown, since none of the two tested baselines were able to reliably detect 5 mm displacement in a high percentage (less than 90%). On the other hand, displacements greater than 20 mm are not presented, because all of them were reliably detected.

Results from Table 9 are based on 270 statistical tests that were done for all possible differences between sessions where displacement was imposed. We may see that displacements of 10 mm and onward are all successfully detected on a basis of 30-min sessions and in favorable conditions. In real situations, we may experience a decrease in the rate of successfully detected displacements due to the impact of obstacles, multipath, bad weather conditions, etc. [11,12].

The 3D case is just a combination of 1D and 2D cases, so the results in Table 10 are very similar to the 2D case. However, a slight drop (3%) in detected displacements for the L-baseline of 10 mm displacement is due to the error in height component (see Table 8).

**Figure 7:**
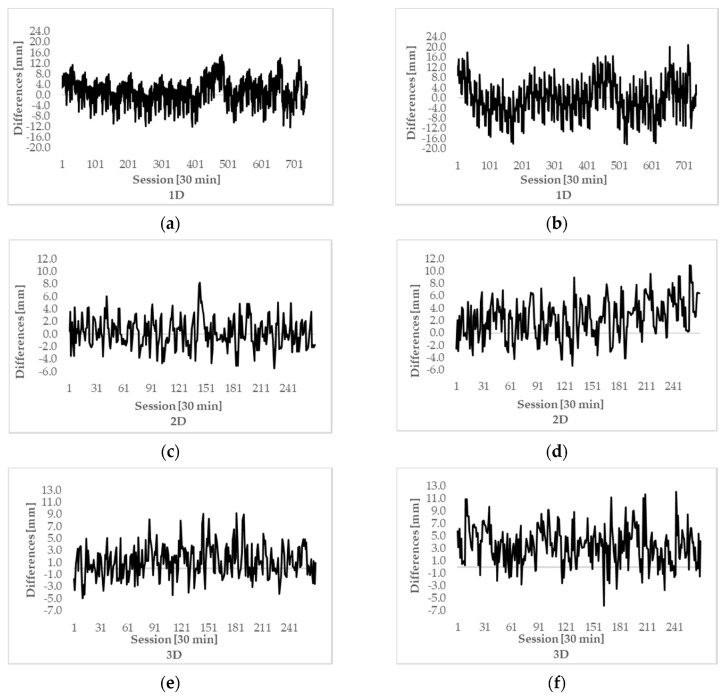
1D, 2D and 3D differences between true and estimated displacement of point FGG1: (**a**) 1D, G-baseline; (**b**) 1D, L-baseline; (**c**) 2D, G-baseline; (**d**) 2D, L-baseline; (**e**) 3D, G-baseline; (**f**) 3D, L-baseline.

Figure 7 represents graphically results from Table 8, Table 9 and Table 10, where differences between true and estimated displacement in all three dimensions, i.e., 1D, 2D, and 3D, are shown. True displacements were always zero for 1D case and in steps of 5 mm for 2D and 3D cases. There are 720 calculated displacements in the case of 1D and 270 calculated displacements for 2D and 3D cases. In 1D cases, the variation in differences is larger, while for 2D and 3D cases, it is slightly smaller.

The last table, Table 11, represents MAE values, the averages of absolute values, calculated for all data from Figure 7. The lowest MAE is for both baselines determined for the 2D case, followed by the 3D case and the highest MAE is determined for the height component (1D). We have to emphasize here that the highest value of MAE is for 1D only because many more observations are used in this case. The number of 1D tests for displacement detection was 720 (static and dynamic test, see Section 2.4.2), whereas, in the 2D and 3D cases, the number of tests was only 270 (dynamic test only). However, we may also see that the G-baseline has a lower level of MAE for all three dimensions. The improvement in accuracy for the G-baseline over L-baseline is 20% for 1D, and 40% for 2D and 3D.

The results from Table 11 show that geodetic instruments had a better performance than low-cost GNSS instruments, despite the presence of antenna offset bias in G-baseline. This may indicate several factors, namely, worse quality of low-cost GNSS receiver, worse quality of low-cost external GNSS antenna, and the possibility that individual antenna calibration parameters should be determined for every low-cost GNSS antenna (type calibration does not represent all individual antennae). However, to fully understand all possible causes, additional tests and analysis must be done in the future. Some possible solutions may be seen in using different external antennae, performing individual relative antenna calibrations, or using an external antenna with known calibration data [14,20].

## 4. Conclusions

In geodetic monitoring, geodetic GNSS receivers and antennae are usually used as they provide high-quality results. However, in cases where there is high risk of instrument damage, these may be regarded as unsuitable. It is then more appropriate to use low-cost GNSS receivers/antennae. To date, those were usually single-frequency receivers, but recently double-frequency, low-cost receivers are also available at the market.

In this paper, we analyzed the positional quality and the potential for displacement detection of a ZED-F9P low-cost GNSS receiver in combination with an ANN-MB-00 low-cost GNSS antenna. The survey was done in favorable conditions where a static as well as dynamic survey was performed. The results obtained in this work lead to the following conclusions regarding the used low-cost equipment:

- Geodetic GNSS receiver with a geodetic antenna and low-cost receiver with the low-cost antenna had slightly better performance but the positional quality of used low-cost GNSS equipment is well enough and accepted in applications such as geodetic monitoring of natural hazards;
- 3D displacements in a range of 10 mm can be detected by using low-cost GNSS instrumentation with a high level of reliability in open sky;
- 2D displacements were estimated with higher accuracy than 3D and 1D displacements, used antennas are non-calibrated and this probably influences the precision of displacements’ estimation, especially the vertical component;
- SimpleRTK2B board is cheap, light, and easily configurable through the freely available u-center application software. Considering these characteristics and the proven performance, it can be classified as a proper sensor in few monitoring applications.

However, it should be noted here that all tests were performed in favorable surveying conditions and with short baselines. We expect that under adverse conditions the results would deteriorate. Therefore, it would be useful to perform additional tests in the future where several factors should be analyzed. One of them is adverse surveying conditions. On the other hand, to eliminate antenna bias, a calibrated GNSS antennae should also be used, with the possibility of individual antenna calibration parameters. Low-cost GNSS instruments should also be analyzed to separately determine the performance of receiver and antenna.

## Figures and Tables

**Table 1:** Used RTKLIB parameters for data processing.

Parameters	RTKLIB
Elevation Angle	10°
Observations	L1, L2
Session Length	1 h, 30 min
Constellations	GPS, GLONASS, Galileo
Troposphere	Saastamoinen
Ephemeris	Broadcast
Ambiguity	Fix and hold (LAMBDA)

**Table 2:** Elementary residual statistics for G-baseline.

Statistics	Y (mm)	X (mm)	h (mm)
Min.	−3.6	−3.2	−9.1
Max.	2.8	4.4	8.2
RMSE	1.5	2.0	4.2

**Table 3:** Elementary residual statistics for L-baseline.

Statistics	Y (mm)	X (mm)	h (mm)
Min.	−2.8	−3.5	−7.7
Max.	4.1	4.3	5.2
RMSE	1.6	2.0	2.9

**Table 4:** Residual classification for G-baseline.

Range	Y	X	h
0–2 mm	86%	54%	32%
2–4 mm	14%	43%	29%
4–6 mm	0%	3%	25%
6–8 mm	0%	0%	7%
8–10 mm	0%	0%	7%

**Table 5:** Residual classification for L-baseline.

Range	Y	X	h
0–2 mm	81%	68%	46%
2–4 mm	16%	26%	36%
4–6 mm	3%	6%	14%
6–8 mm	0%	0%	4%
8–10 mm	0%	0%	0%

**Table 6:** Differences of 1D, 2D, and 3D positions from G-baseline and L-baseline.

Position	1D	2D	3D
Differences [mm]	10.4	2.6	10.7
Differences precision [mm]	4.6	2.5	5.2

**Table 7:** Comparison of low-cost and geodetic GNSS antennas with ANOVA statistical test.

ANOVA	Y	X	h
F	5.27	17.61	110.69
$F_{\mathrm{critical}}$	4.02	4.02	4.02

**Table 8:** Detection of displacement in 1D (height component).

	G-Baseline	L-Baseline
Not Detected	98%	77%
Detected	2%	23%

**Table 9:** Detection of 2D displacements.

	G-Baseline			L-Baseline		
Displacement Size	10 mm	15 mm	20 mm	10 mm	15 mm	20 mm
Detected	100%	100%	100%	100%	100%	100%

**Table 10:** Detection of 3D displacements.

	G-Baseline			L-Baseline		
Displacement Size	10 mm	15 mm	20 mm	10 mm	15 mm	20 mm
Detected	100%	100%	100%	97%	100%	100%

**Table 11:** Mean Absolute Error (MAE) for evaluated 1D, 2D, and 3D movements.

	G-Baseline			L-Baseline		
Dimension	1D	2D	3D	1D	2D	3D
MAE (mm)	4.0	1.9	2.3	5.0	3.2	3.8
